# Raman Spectra of Luminescent Graphene Oxide (GO)-Phosphor Hybrid Nanoscrolls

**DOI:** 10.3390/ma8125470

**Published:** 2015-12-04

**Authors:** Janardhanan. R. Rani, Se-I Oh, Jae-Hyung Jang

**Affiliations:** School of Information and Communications, Gwangju Institute of Science and Technology, Oryongdong, Buk-gu, Gwangju 61005, Korea; ranijnair@gmail.com (J.R.R.); ohseida@gist.ac.kr (S.-.I.O.)

**Keywords:** graphene oxide (GO), nanoscrolls, raman analysis, photoluminescence

## Abstract

Graphene oxide (GO)-phosphor hybrid nanoscrolls were synthesized using a simple chemical method. The GO-phosphor ratio was varied to find the optimum ratio for enhanced optical characteristics of the hybrid. A scanning electron microscope analysis revealed that synthesized GO scrolls achieved a length of over 20 μm with interior cavities. The GO-phosphor hybrid is extensively analyzed using Raman spectroscopy, suggesting that various Raman combination modes are activated with the appearance of a low-frequency radial breathing-like mode (RBLM) of the type observed in carbon nanotubes. All of the synthesized GO-phosphor hybrids exhibit an intense luminescent emission around 540 nm along with a broad emission at approximately 400 nm, with the intensity ratio varying with the GO-phosphor ratio. The photoluminescence emissions were gauged using Commission Internationale d'Eclairage (CIE) coordinates and at an optimum ratio. The coordinates shift to the white region of the color spectra. Our study suggests that the GO-phosphor hybrid nanoscrolls are suitable candidates for light-emitting applications.

## 1. Introduction

Graphene oxide (GO) has received significant attention due to its unique electronic, mechanical, and thermal properties [1,2,3]. Recent efforts have been devoted to exploring the applications of GO in electronic and optical devices due to its tunable optoelectronic properties as well as its excellent electrical, mechanical, and thermal properties [3]. Owing to its excellent wiring with biomolecules, a heterogeneous chemical and electronic structure, and solution processability, GO also finds application in biosensing platforms [4,5]. In pristine graphene, all atoms are *sp*^2^-hybridized, and the zero optical band of the Dirac cone is a hindrance to luminescent emission. Many light-emitting diode applications of graphene have not been realized due to the absence of strong luminescent emissions. However, GO oxide (GO) consists of *sp*^2^ and *sp*^3^ carbon atoms due to the attachment of oxygen, and the ratio of *sp*^2^/*sp*^3^ fractions opens up new possibilities for luminescent emissions [6]. The luminescence output from GO is poor and currently not suitable for practical applications. Highly efficient inorganic phosphors which emit light via photo and electron excitation are widely used in the display industry. Therefore, a GO-phosphor hybrid will have the unique advantages of the luminescent emission from the phosphor along with the superior physical and chemical properties of the GO. Graphene oxide nanoscrolls (GONS) are new members in the GO family, formed through the rolling of GO layers in one or more directions to synthesize novel GO-based carbon materials [7]. GONS are considered as another interesting carbon material and are regarded as defective carbon nanotubes (CNTs), as these structures are distinct from GO layers and multiwall carbon nanotubes [8]. Due to their unique structure and tunable core size, GONS find applications in hydrogen storage, supercapacitors, batteries, nanotransistors, and biosensor devices [9,10,11]. Nanoscrolls of other two-dimensional (2D) materials such as MoS_2_ exhibit a strong photoluminescence, ranging from 420 nm to 600 nm [12].

In the present study, due to the heavy phosphor attachment, GO transforms into scrolls and emits strong luminescence. We report highly luminescent GO-phosphor nanoscrolls which have the advantage of both GO and a luminescent phosphor material [13]. The structural and optical characteristics of the hybrids are analyzed in detail. The mechanisms of scroll formation and photoluminescent emission are also discussed.

Here, we analyze the structure and the formation mechanism of nanoscrolls in detail using Raman spectroscopy. GO-phosphor nanoscrolls were prepared by varying the GO:phosphor ratio. The luminescent emission was tuned by varying the GO:phosphor ratio. We found that by controlling this ratio, Commission Internationale d'Eclairage (CIE) coordinates of the emission shift towards the white region of the spectra. Our GO-phosphor hybrid nanoscrolls are expected to have promising applications in light-emitting diode applications.

## 2. Materials and Methods

Graphite oxide was synthesized using the modified Hummers method [14]. The phosphor material (SrBaSi_2_O_2_N_2_:Eu^2+^) was embedded in a GO solution via a simple chemical process. The GO solution (concentration 0.3 mg/mL) and the phosphor colloid solution (various concentrations of 0.05, 0.07, 0.1, 0.125, 0.15 mg/mL) were separately sonicated in deionized water for 30 min, after which they were mixed and further sonicated for 10 min. The substrates were cleaned initially with distilled water and then in an ultrasonic cleaner in an acetone bath for 30 min. The GO-phosphor hybrid solutions were spin-casted onto standard Si/SiO2 substrates at 500, 800, and 1600 rpm for 30 s and were then annealed at 160° for 5 min. The films were termed GO (undoped), GNP1, GNP2, GNP3, GNP4, and GNP5 (phosphor-attached GO film with concentrated solutions at 0.05, 0.07, 0.1, 0.125, and 0.15 mg/mL, respectively). The structural and optical changes are monitored by scanning electron microscopy (SEM) (JEOL, JSM-6700F, Tokyo, Japan), atomic force microscopy (AFM) (multimode, Veeco, New York, NY, USA), Raman spectroscopy (inVia Raman microscope, Renishaw, Gloucestershire, UK), and photoluminescence (PL) (Perkin-Elmer LS 55, Perkin Elmer, Waltham, MA, USA) measurements.

## 3. Results and Discussions

**Figure 1:**
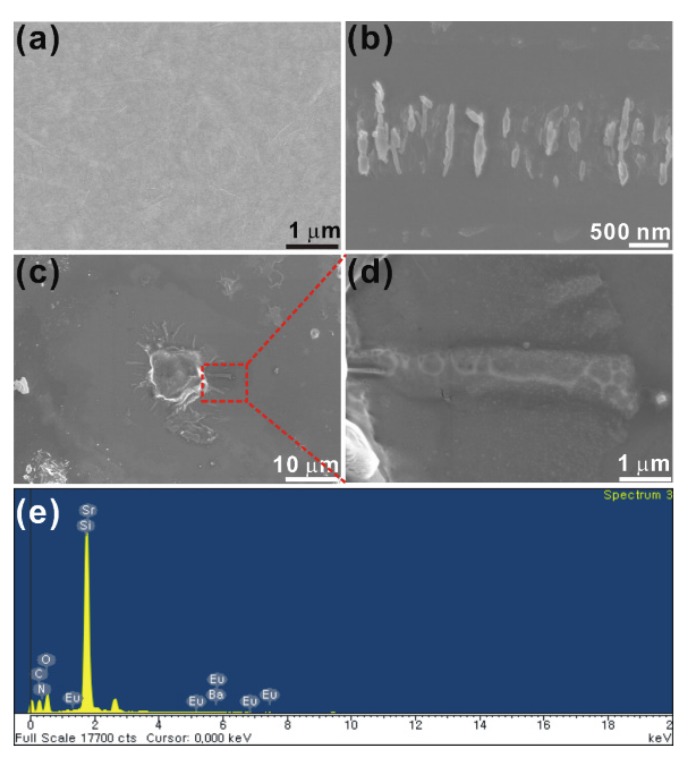
SEM images of (**a**) GO; (**b**,**c**) various regions of GNP1 film; (**d**) an expanded view of the single scroll shown in the red dotted square in (**c**); and (**e**) the EDAX spectra from the scroll.

Figure 1 shows scanning electron microscopy (SEM) images of the films. The pristine GO film does not show any scrolls (Figure 1a). Figure 1b,c show SEM images of various regions of the GNP1 film, providing a clear image of the formation of the nanoscrolls. Figure 1d shows an expanded view of the single scroll shown in Figure 1c. The composition of the scrolls was analyzed using energy dispersive X-ray spectroscopy analysis and the corresponding spectra from the scroll shown in Figure 1d are depicted in Figure 1e.

In the GNP1 films, the edges of the GO layer fold back and scroll into tubular structures, resulting in the formation of nanoscrolls. Upon sonication of the GO phosphor in water, the phosphor particles become attached onto GO sheets, spontaneously folding the GO flakes. The driving force of scrolling is the energy difference between the total surface energy of the system and the elastic energy associated with GO bending [15]. X-ray photoelectron spectroscopy (XPS) analyses of our films confirmed the presence of C–N bonding. Raman studies of our films also provided evidence of C–N bonding. Thus, during the annealing process, phosphor particles become attached to the flat GO sheets via C–N bonding. When the temperature increases, the GO sheets bend around the phosphor particles in order to minimize the total surface energy of the GO-phosphor system by reducing the exposed surfaces of both the GO and the phosphor. Second, after the phosphor particle is wrapped by a GO sheet, the subsequent rolling of GO is driven by the reduction of the total area of the exposed GO surface. Consequently, the total surface energy of the system is minimized by the tightly wrapping adjacent layers of the scroll.

**Figure 2:**
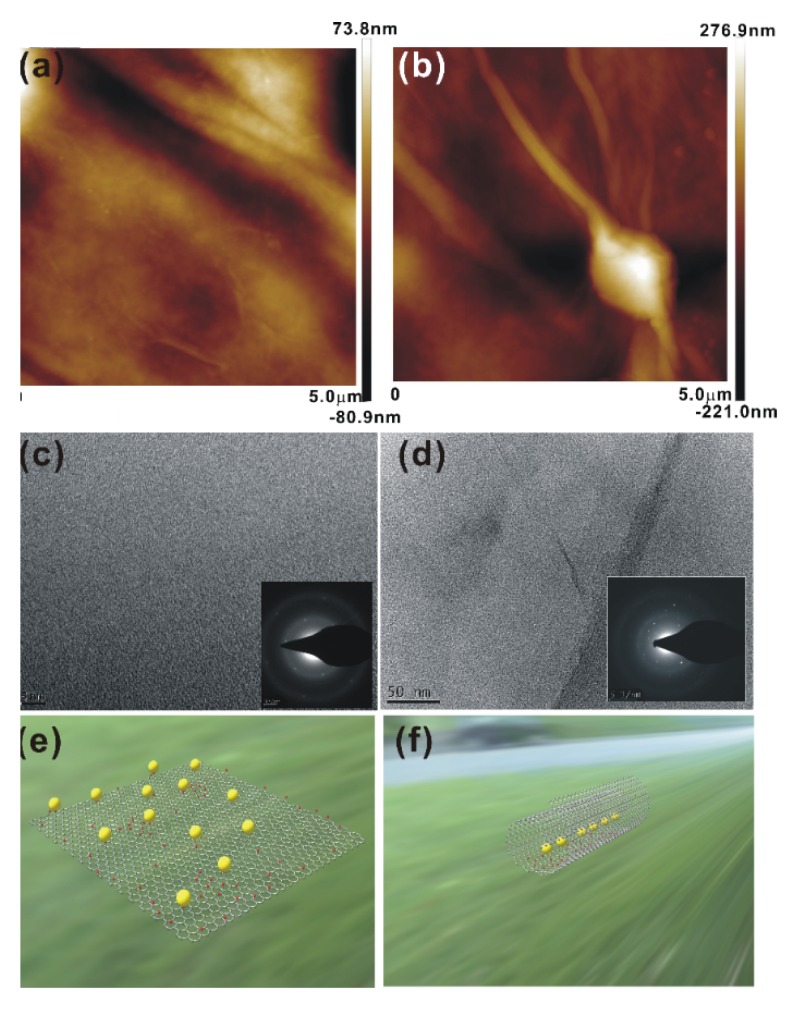
(**a**,**b**) AFM images; and (**c**,**d**) TEM images of GO and GNP1 films, respectively: The inset figures in (**c**,**d**) show the corresponding SAED patterns; and (**e**,**f**) show schematic diagrams of phosphor particles attached to GO sheets and completely scrolled sheets, respectively.

The AFM images of the GO and GNP1 films shown in Figure 2a,b also confirm the formation of nanoscrolls in GNP1 films. Figure 2c,d show TEM images of the GO and GNP1 films, and the inset figures show the corresponding selected area electron diffraction (SAED) patterns. The SAED pattern of the GO film depicts the (002) reflections of the GO lattice [16]. The SAED pattern of the GNP1 film shows a mixed pattern of GO and CNT. Figure 2e,f show schematic diagrams of phosphor particles attached to GO sheets and completely scrolled sheets, respectively.

**Figure 3:**
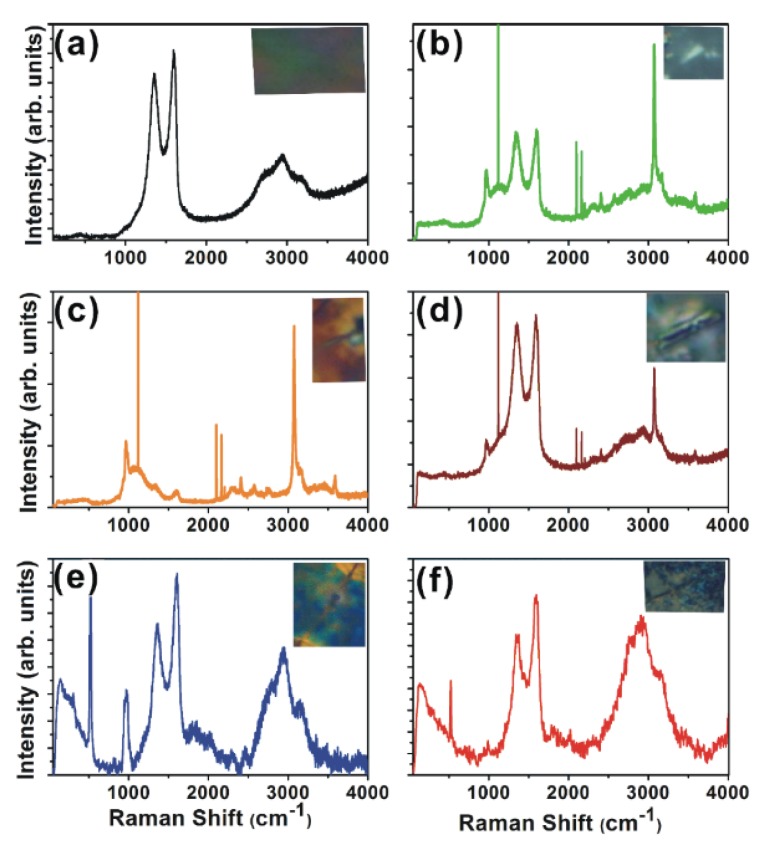
Raman spectra of (**a**) GO; (**b**–**f**) different regions of GNP1 film. The corresponding optical images are shown in the inset of each figure.

For a deeper examination of the nanoscrolls, Raman analyses were performed. Figure 3a shows a Raman image of the GO film, and Figure 3b–f show Raman images of the GNP1 film in different regions. The corresponding nanoscrolls are shown in the inset figures. The GO and GNP1 films show spectral features of GO such as peaks at 1590 cm^−1^ (G peak), 1350 cm^−1^ (D peak), 2697 cm^−1^ (2D peak), and at ~2940 cm^−1^ (G + D peak) [17,18,19]. The *E*_2g_ vibrational and out-of-plane modes within aromatic carbon rings result in the formation of the G and 2D bands, respectively. The G band is a degenerated optical phonon mode at the Brillouin zone center and is induced by a single resonance process, while the D band requires scattering at defect sites in order to conserve momentum. It was found that the broadened D band in scrolled GO arises due to the conservation of the momentum of the intra-valley electrons scattered via iTO phonons (at the *K*-point) by curvature-induced defect scattering [20]. The Raman spectrum of the scrolled GO differs significantly from that of flat GO. The high degree of curvature in the scrolled GO causes the appearance of low-frequency radial breathing-like (RBLM) modes.

The most important feature in the Raman spectrum of CNTs is the radial breathing mode (RBM), which is usually located between 75 cm^−1^ and 300 cm^−1^ [20]. Figure 3b–f show RBM modes around 140 cm^−1^ and Figure 3e,f show additional radial breathing mode (RBM) features around 290 cm^−1^. In addition to SAED patterns, the Raman spectra confirm the tubular structure of the GO scrolls. The RBLM peak intensity varies in different scrolls and is found to be less intense where no perfect scroll is formed. Thus, the RBLMs provide strong support for scrolled GO. The radial breathing mode is absent in the Raman spectrum of the flat GO (Figure 3a), as the low-frequency vibrations correspond to a simple translation of the honeycomb network. However, the curvature in the scroll transforms this translation into a phonon mode, similar to the radial breathing mode in CNTs.

Figure 3b–f show peaks in the range of 910–1050 cm^−1^, corresponding to the iTA phonons reported for graphite whiskers and CNTs [21]. This peak is absent in pure GO, and its appearance is due to the scrolling of the GO layers. The perfectly scrolled tubular structure (Figure 3e–f) shows a peak around 1800 cm^−1^ and 2000 cm^−1^. Out-of-plane phonons around the Γ point are relevant to these weak Raman peaks. The double-resonance Raman scattering process occurs during the intra-valley scattering process, and the combined modes of LA + iTA (iTALO+) corresponding to the inter-valley scattering process appear in the range of 1800–2000 cm^−1^ [22]. Thus, the peak at 1800 cm^−1^ is a combination of iTA and LO phonons, *i.e.*, iTALO. The combination of the oTO (out-of-plane tangential optical) + LO phonon mode around the K point in the GO Brillouin zone presents a peak around 2000 cm^−1^. Due to the odd symmetry for the mirror operation on a single-layer GO plane, the oTO phonon at the Γ point is not a Raman active mode and, thus, the overtone of the oTO phonon is not observed for mono-layer graphene. The out-of-plane tangential optical (oTO) phonon of single-layer graphene at the Γ point in the two-dimensional Brillouin zone is not a Raman active mode due to the odd symmetry for the mirror operation on a GO plane. On the other hand, the Raman band of the overtone of the oTO phonon of single-walled carbon nanotubes (SWNTs) is observed and is known as the M band, as the cylindrical shape of the SWNTs activates the M-band Raman spectra. Thus, for a perfectly scrolled tubular structure, these modes are active due to the resemblance with CNTs.

The *sp*^2^-bonded C–N groups give rise to stretching modes around 2200 cm^−1^ and are observed in the GNP1 film, which confirms the bonding between the carbon and nitrogen. Another mode observed at ~2280 cm^−1^ is a combination of the iTO and iTA phonons (the iTOTA mode) around the *K* point and is activated in GNP1 film, even if it is not perfectly scrolled [20]. A combination of the zone boundary in-plane longitudinal acoustic (iLA) phonon and the in-plane transverse optical (iTO) phonon modes results in a G* band around 2450 cm^−1^ which is also activated in GNP1 film.

**Figure 4:**
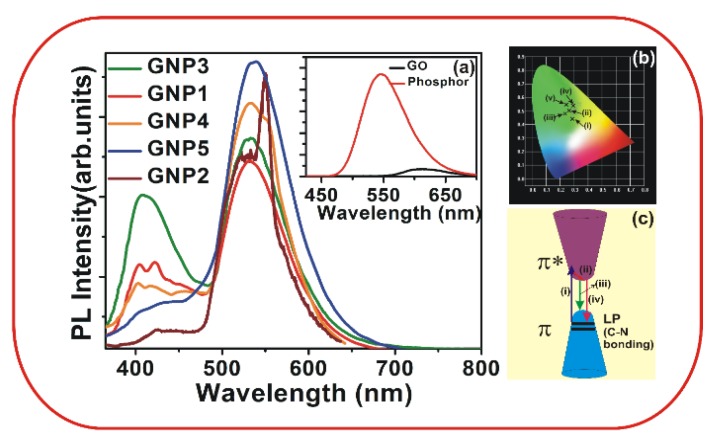
PL emission spectra of (**a**) the GNP1-5 films at various phosphor concentrations as measured with an excitation wavelength of 280 nm. The inset shows the PL emission from pure GO and the reference phosphor; (**b**) CIE chromaticity coordinates of the PL emission from the synthesized thin films; (i), (ii), (iii), (iv) and (v) correspond to GNP3, GNP1, GNP4, GNP5 and GNP2, respectively; and (**c**) presents a schematic of the PL emission from the films.

Figure 4a shows the photoluminescence (PL) emission spectra of films prepared with different phosphor concentrations, and Figure 4b shows the 1931 CIE chromaticity coordinates of the PL emission from the synthesized thin films. The reference phosphor exhibits strong a emission around 537 nm which is due to the emission from Europium (Eu^2+^) levels in the phosphor (inset of Figure 4a) [23,24]. All of the films show a broad peak around 400 nm, an intense peak around 537 nm, and weak emission in the visible near-IR (VIS-NIR) region. The peak at 537 nm is from the phosphor, while the weak emission around 620 nm is due to the bandgap emission of GO (inset of Figure 4a). Typically, the PL emission in pure GO is very weak around 530 nm and is due to the emission from the π–π* energy gap. The π–π* energy gap is formed due to the disruption of π networks which result from the attachment of oxygen functional groups onto the basal plane of GO. Reports show that GO films exhibit broad luminescent emissions in the UV-Visible region; these films also show blue emissions centered around 390−440 nm. The blue PL emission is the radiative recombination of electron−hole pairs (e−h pairs) generated within localized states; the broad PL was believed to originate from the carbon *sp*^2^ domains/clusters. In our case GO exhibits weak luminescence around 620 nm (inset of Figure 4a).

The intense peak around 537 nm is due to the emission from the phosphor material. Because the bandgap emission from GO is very weak, merging takes place with the intense peak around 537 nm.

The broad emission around 400 nm is due to C–N bonding, which we also observed in the Raman spectra. Usually, in CN films, photoluminescence in the visible region mainly occurs due to transitions between the lone pair (LP) valence band and the π* conduction band. The LP state is formed because the LP electrons of nitride are not hybridized with the carbon and it is located in the *sp*^2^ C–N π valence band. The transition from the π* state to the LP state results in the emission around 400 nm.

Figure 4c shows a schematic representation of these emission peaks. The arrow marked as (i) in Figure 4c corresponds to the absorption of the photon; (ii) corresponds to the non-radiative transition; (iii) represents the bandgap emission in GO; and (iv) represents the emission at around 400 nm. The PL emission changes with the concentration of the phosphor, as shown in Figure 4a. The concentration of the phosphor increases from GNP1 to GNP5; however, the emission at 400 nm does not increase continuously. The GNP3 film shows maximum intensity at 400 nm. The order of variation in the 400 nm emission intensity is GNP3 > GNP1 > GNP4> > GNP5 > GNP2. As the phosphor concentration increases, the C–N bonding and the corresponding probability of a transition between the π* band and the LP electron level increases, which in turn results in an increase in the emission intensity around 400 nm up to GNP3. However, a further increase in the phosphor concentration results in the re-absorption of the emitted photons, and this process reduces the emission intensity of the 400 nm light. Thus, the intensity of the emission around 400 nm varies with the phosphor concentration. The intensity around 400 nm is higher for the GNP3 film and lower for the GNP1 film, as shown in Figure 4b. The CIE coordinates show that the PL emission for the GNP3 film shifts towards the white region.

## 4. Conclusions

We report a facile synthesis of GO oxide nanoscrolls using a simple chemical method. The SEM and HRTEM images of the GNP1 film show scrolls, and the lattice planes measured from the SAED pattern exhibit CNT-like structural ordering. We found that the Raman spectrum of the scrolled GO differs significantly from that of the flat GO. Specifically, the appearance of a broadened D band in the scrolled GO is due to the momentum conservation of the intra-valley electrons. The GO-phosphor hybrids show a highly intense luminescence emission around 540 nm. The C–N bonding results in an additional broad luminescent emission at approximately 400 nm, and this peak intensity varies with the phosphor concentration. The emission from the GNP3 film shifts towards white and is expected to be useful for important applications in the fabrication of GO-based optoelectronic devices.
